# Protocol for the Development of a Food Stress Index to Identify Households Most at Risk of Food Insecurity in Western Australia

**DOI:** 10.3390/ijerph16010079

**Published:** 2018-12-29

**Authors:** Timothy J. Landrigan, Deborah A. Kerr, Satvinder S. Dhaliwal, Christina M. Pollard

**Affiliations:** School of Public Health, Curtin University, Kent Street, GPO Box U1987, Perth 6845, Australia; D.Kerr@curtin.edu.au (D.A.K.); S.Dhaliwal@curtin.edu.au (S.S.D.); C.Pollard@curtin.edu.au (C.M.P.)

**Keywords:** food insecurity, food stress, food affordability

## Abstract

Food stress, a similar concept to housing stress, occurs when a household needs to spend more than 25% of their disposable income on food. Households at risk of food stress are vulnerable to food insecurity as a result of inadequate income. A Food Stress Index (FSI) identifies at-risk households, in a particular geographic area, using a range of variables to create a single indicator. Candidate variables were identified using a multi-dimensional framework consisting of household demographics, household income, household expenses, financial stress indicators, food security, food affordability and food availability. The candidate variables were expressed as proportions, of either persons or households, in a geographic area. Principal Component Analysis was used to determine the final variables which resulted in a final set of weighted raw scores. These scores were then scaled to produce the index scores for the Food Stress Index for Western Australia. The results were compared with the Australian Bureau of Statistics’ Socio-Economic Indexes for Areas to determine suitability. The Food Stress Index was found to be a suitable indicator of the relative risk of food stress in Western Australian households. The FSI adds specificity to indices of relative disadvantage specifically related to food insecurity and provides a useful tool for prioritising policy and other responses to this important public health issue.

## 1. Introduction

The different Socio-Economic Indexes for Areas (SEIFA) developed by the Australian Bureau of Statistics (ABS) measure various aspects of socio-economic status. The four different indexes measure relative socio-economic disadvantage (IRSD), relative socio-economic advantage or disadvantage (IRSAD), education and occupation (IEO), and economic resources (IER) [1], however, none of those indexes provide a suitable measure of food insecurity or food stress. The variables used in constructing these indexes are wider socio-economic measures and don’t relate specifically to food insecurity. In order to measure food insecurity, these indexes need to be used in conjunction with other data such as food costs to provide an indication of the impact of socio-economic status and food costs on health [2,3,4,5,6].

The concept of a Food Stress Index (FSI) is to provide a simple indication of the potential for food stress of households in a particular geographic location which may be postcode, Statistical Area, Local Government Area or another region. It is a single index that encompasses all aspects of food insecurity to provide information about the likelihood households in a geographic area are suffering food stress.

Housing stress is usually defined as occurring for those households who spend more than 30% of their income on housing costs, whether that is rent or mortgage [7]. This is particularly critical for those households whose income is in the 1st or 2nd quintile. Housing affordability relates to a household’s or a person’s ability to pay for their housing. The impacts of housing stress are widespread as this impacts a household’s spending patterns and has wider effects on the economy as a whole.

Food stress is a similar concept to housing stress and occurs when a household needs to spend more than 25% of their disposable income on food [8]. Australian research has shown that welfare-dependent and low-income households are suffering food stress [9,10,11]. Between 2008 and 2012, this food inequality has risen in Australia [12].

Households at risk of food stress are vulnerable to food insecurity as a result of inadequate income. Food security is “when all people, at all times, have physical and economic access to sufficient, safe and nutritious food to meet their dietary needs and food preferences for an active and healthy life.” [13]. The 2011–2012 Australian Health Survey (AHS) found that four per cent of all Australian households ‘ran out of food in the last 12 months and couldn’t afford to buy more’, increasing to seven per cent of households in the most disadvantaged areas, compared to only one per cent in the least disadvantaged areas. The prevalence was higher among Aboriginal and Torres Strait Islander households with 22% overall and 31% of those households in remote areas running out of food in the previous year [14]. Research looking at the relationship between food security status and multiple socio-demographic variables found that 36% of survey participants had low or very low food security [15].

The United States Department of Agriculture has a Food Security Survey Module (FSSM) [16,17] that is run every two years to measure levels of food security in the United States. In Australia the only routine food security measure is the single-item question on the AHS which does not effectively measure levels of food insecurity [18]. There is no measure, equivalent to the USDA FSSM, that allows routine monitoring of the prevalence of food insecurity at a population level in Australia with the only research to date being undertaken in small geographic areas using small samples [15,18].

Food affordability is defined as the amount of money a household spends on food, relative to income of that household. It is of greater concern for lower income households as they spend a greater proportion of their income on food [19]. Food affordability impacts not only the wider economy through the impacts on spending patterns, but also on health by affecting the ability to purchase healthy and nutritious food [20,21]. While some household expenses are fixed (e.g., rent or utility expenses), the food budget is changeable and can be cut back if needed with nutrition consequences [22]. If one household expense increases (e.g., an increase in rent) then this will impact on that household’s food affordability leading to additional stress on the food budget; i.e., food stress. As a household becomes food stressed, they become vulnerable to food insecurity as they have less available income to meet their dietary needs.

The Food Stress Index is designed as a single measure, using currently available data without the need for additional and expensive surveillance, that ranks geographic areas based on the likelihood that households in those areas are food stressed. The FSI is not a measure of food insecurity as not every household in geographic areas at high risk of food stress would be food insecure; the FSI shows particular geographic areas where households would be more vulnerable to food insecurity. A FSI could be used to measure the impact food affordability has on chronic disease such as diabetes and cardiovascular disease. It could also be used to highlight areas or households in need of food relief.

## 2. Materials and Methods

### 2.1. Developing a Food Stress Index

The methodology used to create the Food Stress Index is similar methodology to that used to develop the Australian Bureau of Statistics’ SEIFA [1] index. The FSI is a weighted combination of select variables that results in a score that can be used to rank areas according to the likelihood of food stress in each area. The index is assigned to areas and reflects the characteristics of the households of people living in an area.

Starting with a broad list of potential or candidate variables covering all aspects of food stress and socio-economic indicators, Principal Component Analysis [23] was used to reduce these variables to a single index which indicates the likelihood households in the selected geographic area are suffering food stress. Low index scores indicate less likelihood of food stress while high index scores indicate more likelihood of food stress. The methodology is discussed in detail below.

### 2.2. The List of Candidate Variables

In order to encapsulate various aspects of food affordability and food stress a wide range of candidate variables were considered when constructing the Food Stress Index. This resulted in an initial set of over 50 candidate variables from eight different datasets from existing surveys. The framework for the selection of variables was based around the following dimensions:

- Household demographics
- Household income
- Household expenses
- Financial stress
- Food affordability
- Geographic information.

Table 1 outlines the dimensions and candidate variables that were considered when developing the index. At this stage, no consideration was made to the availability of the data, only what variables ideally would be suitable when constructing a Food Stress Index. Final decisions on the availability and suitability of the candidate variables were made in the next step.

The initial set of candidate variables was reduced before constructing the final index. When reducing the variables to a more manageable set, consideration was made of the suitability of each variable, and the potential to match variables by the selected geography, in this case the ABS’ geographic classification of Statistical Area 2. This meant that variables that were not available on the same geographic basis for the respective households were not considered further. It is anticipated that the index will be regularly updated, and it was important that data was available from within the last five years to maintain relevance. Census data met both of these considerations and was the preferred source of data. In the case of the Food Access and Cost Survey (FACS) [26], the most recent data was from 2013 and the 2016 Census of Population and Housing [24] was used for all other variables.

From the list of variables in Table 1, household expense variables and financial stress indicators weren’t available within the five-year period, or at the desired geography for this work. The sources for these variables are irregular surveys run by the ABS; as a result these variables were excluded from the Food Stress Index.

### 2.3. Description of Variables Used

The variables relate to persons, families or households and were expressed as a proportion of units in an area with the specified characteristic. Each of the dimensions is discussed below.

#### 2.3.1. Household Demographics

Household composition, including family size, the number of parents and the age of any children provides a good indicator of household size, income and expenses. A single parent household with children under the age of 15 will have more difficulty earning income and meeting weekly expenses as they are generally able to only work on a part-time or casual basis due to child care commitments [27]. Single parent households also have to spend a greater proportion of their income to purchase a healthy meal plan [19].

The 2016 Australian Census shows that 45% of families were families with two parents and children while 38% of families were couples without children, and 16% were single parent families [24]. Using this information, the variables selected to demonstrate most, and least likelihood of food stress are the proportions of single parent or two parent households, with or without children under 15, within the selected geographic area.

#### 2.3.2. Indigenous Status

The Indigenous status of a household provides a strong indicator of whether or not that household is likely to suffer from food stress. There are high unemployment rates and low income among Aboriginal and Torres Strait Islander peoples as well as significant disparities in health status between Aboriginal and Torres Strait Islander peoples and other Australians [28]. The proportion of Indigenous households from the 2016 Census was used.

#### 2.3.3. Household Income

Income is an important indicator of the likelihood of food stress with households in the lowest income quintiles needing to spend a higher proportion of the income on food than those households in the highest income quintiles [19]. The income variable used was the Equivalised Total Household Income variable from the 2016 Census. Equivalised household income is household income which has been adjusted by an ‘equivalence scale’ based on the number of adults and children in the household [1].

Low income was defined as the proportion of households in the first quintile of the equivalised household income distribution; i.e., those households earning between $1 and $25,999 per year. These households represent those most likely to suffer food stress. The households least likely to suffer food stress were defined as those in the top income quintile, earning more than $78,000 per year.

#### 2.3.4. Food Affordability

Food costs, as measured by the proportion of income required to purchase a healthy meal plan for a household, vary depending on the income of the household. Households that need to spend more than 25% of their income on food are suffering food stress [8] and the proportion of income required provides the strongest indicator of food stress.

Data from the 2013 FACS [26] was used to estimate food affordability; i.e., the proportion of income required to purchase a healthy meal plan for households of different compositions and incomes.

#### 2.3.5. Geographic Information

Most of the data was taken from ABS datasets so each of the variables was available on ABS geography and Statistical Area 2 (SA2) was used as the base geography. Data from the FACS was also available by SA2s. Various SA2s were excluded from the list of SA2s because of the type of area (e.g., national parks, airports and industrial areas) or there was insufficient data (i.e., two or more variables unavailable). When the invalid areas were removed, 228 SA2s remained of the 253 SA2s in Western Australia. Data from the 2013 FACS was only available for 76 SA2s so the Food Stress Index was created for these SA2s.

### 2.4. Reduced Set of Variables

The final set of 13 variables selected is shown in Table 2.

Once the initial variables were identified, Principal Component Analysis (PCA) [23] was used to create the Food Stress Index. The PCA technique summarises a number of correlated variables into a set of new uncorrelated components to allow for easier analysis. By removing correlated variables, the technique reduces the number of variables to a set that summarises the information and enables easier analysis. The PCA process results in a set of weighted raw scores that can then be scaled to produce the index scores for the Food Stress Index.

#### 2.4.1. Create Proportions for the List of Initial Variables

Each variable was created as a proportion of units within the selected geography. For household composition, this was the proportion of families within the area. For the household income and Indigenous status variables, the proportion of households was used. For the food affordability variables, the proportion of income was used.

Each variable was then standardised to a mean of 0 and a standard deviation of 1 using R [29].

#### 2.4.2. Create Correlation Matrix

The correlation matrix was calculated, and highly correlated variables were removed to avoid over-representation of food stress. When two variables measuring conceptually similar aspects of food affordability or food stress had a correlation coefficient of 0.8 in absolute value, one of them was removed.

#### 2.4.3. Conduct Initial PCA

Next, Principal Component Analysis was conducted on the reduced set of variables to obtain the loadings for each variable on the first principal component. Any variables with resulting loadings less than 0.3 were removed on the grounds they were not strong enough to indicate food stress. The PCA step was then repeated until there were no variables with loadings less than 0.3 in absolute value. This resulted in a reduced set of variables, with at least one variable in each of the dimensions covering the food stress and food affordability measures.

### 2.5. Calculate and Scale the Index

Once there was a reduced set of variables, the final step was to calculate the final index. For each SA2, each standardised variable was multiplied by its weight, then summed across all variables. The weight was obtained by dividing the loading for each variable by the square root of the eigenvalue. In order to ensure that low scores indicate least likely to suffer food stress, and high scores indicate most likely to suffer food stress, the sign (positive or negative) for each indicator was set accordingly. That is, indicators of high food stress were given positive signs and indicators of low food stress were given negative signs.

This resulted in scores for each SA2. See the formula below.

$$Z_{SA2}=\overset{p}{\underset{j=1}{\sum }}\frac{L_{j}}{\sqrt{\lambda }}\times v_{j,SA2}$$

where

$Z_{SA2}$ = raw score for the SA2
$v_{j,SA2}$ = standardised variable of the *j*-th variable for the SA2
$L_{j}$ = loading for the *j*-th variable
λ = eigenvalue of the principal component
p*=* total number of variables in the index

To create a meaningful index, the scores were scaled with a mean of 1000 and standard deviation of 100 to create a new set of scores ranking the SA2s in order from least likely to suffer food stress to most likely to suffer food stress.

## 3. Results

The Food Stress Index was created for 76 SA2s in Western Australia. The scores ranged from 873.5 for North Perth (which is in the most advantaged SEIFA quintile), to 1400.4 for Halls Creek (in the most disadvantaged SEIFA quintile). This meant that households in the inner Perth suburb of North Perth are the least likely to suffer food stress in Western Australia and households in the remote north-west town of Halls Creek are most likely to suffer food stress. Table 3 shows the SA2s in each quintile of the Food Stress Index.

The Food Stress Index scores were compared with SEIFA Index of Relative Socio-economic Advantage and Disadvantage (IRSAD) for consistency. For example, the IRSAD for Mt Hawthorn/Leederville falls in the tenth decile, meaning persons living there are most advantaged. This aligns well with the Food Stress Index for Mt Hawthorn/Leederville which falls in the first decile, meaning persons living there have the least likelihood of food stress. To test the suitability of the FSI, a Spearman’s correlation was run to determine the relationship between the Food Stress Index and the IRSAD index. There was a strong, negative correlation with the IRSAD index (*r* = −0.89, *p* < 0.001).

## 4. Discussion

The Food Stress Index provides a measure of the likelihood that households in a geographic area are vulnerable to food stress. When applied to Statistical Area 2 (SA2), households in the more remote areas of Western Australia are most likely to suffer food stress (e.g., East Pilbara, Halls Creek, Kununurra). Households in Perth metropolitan areas are least likely to suffer from food stress (e.g., North Perth, Mount Hawthorn and Ocean Reef). The FSI provides more information on food security than the widely used SEIFA which measures socioeconomic status. For example, although Ashburton is in a remote part of Western Australia and is in the third quintile for SEIFA, the FSI takes into account the high proportion of households in Ashburton that are in the highest income quintile and the low proportion of single parent families, resulting in a low Food Stress Index. Similarly, within the Perth metropolitan area, households in Girrawheen, are more likely to suffer food stress due the high proportion of households in the lowest income quintile and the high proportion of Indigenous households.

One of the limitations of this research was that some of the candidate variables (i.e., household expense and financial stress data) were not available at the required level of detail when the analysis was undertaken. Although this data wasn’t included it was still possible to construct a suitable index with the available data. Further research is planned to determine the implications of including this data if it becomes available.

## 5. Conclusions

The Food Stress Index, the first of its kind in Australia, is a suitable indicator of the risk of food stress in Western Australian households. It incorporates a range of variables to measure food stress including food costs, household composition and household incomes. Further research is needed to develop the FSI methodology for smaller geographic areas such as Statistical Area 1 (SA1) to be more representative of households. Population weighted averages of the SA1s would be used to construct indexes for larger geographies. The FSI could be applied to all Australian households, providing a useful tool for national food security. The FSI can be used for to highlight areas where households are more likely to be food stressed and more vulnerable to food insecurity. Policy and intervention planning can then better target services to where they are needed.

## Figures and Tables

**Table 1:** Candidate variables for a Food Stress Index.

Dimensions	Description of Measure	Description of Candidate Variables	Data Source
Household demographics	Proportion households by family composition	Couple families with children under 15 Single parent families with children under 15 Couple families with no children under 15 Single parent families with no children under 15	ABS: 2016 Census, Datapacks, General Community Profile, Western Australia [24]
Aboriginal and Torres Strait Islander Peoples	Proportion of Aboriginal and Torres Strait Islander households	Indigenous status	ABS: 2016 Census, Datapacks, General Community Profile, Western Australia [24]
Household income	Income quintiles of household	Proportion of households in the lowest income quintile Proportion of households in the highest income quintile	ABS: 2016 Census, Tablebuilder, Counting Persons, Place of Enumeration, Equivalised Total Household Income (weekly) [24]
Household expenses	Proportion of income used for household expenses (excluding food)	Housing costs (rent/mortgage) Transport Utilities Education	ABS: Household Expenditure Survey [25]
Financial stress indicators	A measure of whether households may be experiencing economic hardship, based on how many of the financial stress indicators a household experiences.	Financial stress experiences (e.g., unable to raise funds for an emergency, unable to pay bills on time) Missing out experiences (e.g., could not afford a holiday for at least a week, could not afford a special meal once a week)	ABS: Household Expenditure Survey [25]
Food affordability and access	Food affordability for the household. Access to food for the household.	Proportion of income required to purchase a healthy meal plan. Number of supermarkets within geographic area as an indication of access to affordable food.	2013 Food Access and Cost Survey [26]

**Table 2:** Reduced set of variables used to construct the Food Stress Index.

Dimensions	Description
Household demographics	Proportion of couple families with no children under 15
	Proportion of couple families with children under 15
	Proportion of one parent families with no children under 15
	Proportion of one parent families with children under 15
Aboriginal and Torres Strait Islander Peoples	Proportion of Aboriginal and Torres Strait Islander households
Household income	Proportion of households in the lowest equivalised household income quintile (i.e., less than $500 per week)
	Proportion of households in the highest equivalised household income quintile (i.e., more than $1499 per week)
Food affordability	Proportion of income required to buy healthy food–couple family on welfare income
	Proportion of income required to buy healthy food–couple family on low income
	Proportion of income required to buy healthy food–couple family on average income
	Proportion of income required to buy healthy food–one parent family on welfare income
	Proportion of income required to buy healthy food–one parent family on low income
	Proportion of income required to buy healthy food–one parent family on average income

**Table 3:** Food Stress Index for Statistical Areas in Western Australia by quintile, ranging from 1 (least likelihood of food stress) to 5 (most likelihood of food stress).

Food Stress Index Quintile	Western Australia Statistical Areas
1	Applecross—Ardross, Ashburton, Baldivis, Booragoon, Greenwood—Warwick, Innaloo—Doubleview, Karratha, Mount Hawthorn—Leederville, Murdoch—Kardinya, Newman, North Perth, Ocean Reef, Subiaco—Shenton Park, Success—Hammond Park, Wembley—West Leederville—Glendalough, Wembley Downs—Churchlands—Woodlands
2	Australind—Leschenault, Belmont—Ascot—Redcliffe, Bentley—Wilson—St James, Byford, Carramar, Coolbellup, Craigie—Beldon, Eaton—Pelican Point, Esperance Region, Kalgoorlie, Margaret River, Murray, Rivervale—Kewdale—Cloverdale, South Bunbury—Bunbury, Thornlie
3	Albany, Augusta, Busselton, Capel, Denmark, East Bunbury—Glen Iris, Esperance, Geraldton—North, Gingin—Dandaragan, Gnowangerup, Harvey, Maddington—Orange Grove—Martin, Manjimup, Pinjarra, Rockingham
4	Alexander Heights—Koondoola, Beckenham—Kenwick—Langford, Bridgetown—Boyup Brook, Broome, Dowerin, Exmouth, Kambalda—Coolgardie—Norseman, Kulin, Merredin, Moora, Mukinbudin, Narrogin, Northam, Pemberton, Roebourne
5	Armadale—Wungong—Brookdale, Calista, Carnarvon, Cooloongup, Derby—West Kimberley, East Pilbara, Geraldton, Girrawheen, Gosnells, Halls Creek, Kununurra, Leinster—Leonora, Meekatharra, Parmelia—Orelia, Plantagenet, Roebuck
